# Therapists on the front lines: constructing continuous traumatic stress as a dynamic axis of exposure

**DOI:** 10.3389/fpsyt.2026.1888244

**Published:** 2026-08-19

**Authors:** Becky Leshem

**Affiliations:** Department of Education and Leadership, Achva Academic College, Yenon, Israel

**Keywords:** burnout, compassion fatigue, continuous traumatic stress, coping, post-traumatic growth, resilience, shared trauma

## Abstract

The study explored how frontline therapists living and working under ongoing security threats constructed Continuous Traumatic Stress (CTS) and how this experience differed from classical trauma responses. Using a qualitative design, the research drew on 12 semi-structured interviews and a focus group with eight additional mental health professionals in southern Israel, including psychologists, social workers, psychotherapists, and educational counselors exposed directly or indirectly to the October 7, 2023, attacks and their aftermath. Data were analyzed using reflexive thematic analysis and interpreted within a CTS framework. Five interconnected themes emerged: continuous multi-channel exposure, multisystem responses, moral injury, long-term coping trajectories between burnout and renewal, and therapist-specific experiences of secondary traumatization and professional identity crisis. Participants described life in a perpetual “waiting room” state, marked by unrelenting alarms, client trauma narratives, hypervigilance, exhaustion, emotional numbing, and somatic strain. Moral injury was expressed through guilt over interrupting treatment, perceived betrayal of professional values, and distrust in institutional support. Coping fluctuated between chronic burnout and resilience through meaning-making, rituals, and collegial support. CTS was used as a conceptual lens to examine participants’ narratives rather than as a directly measured diagnostic category. The findings suggest a CTS-informed understanding of frontline therapists’ experiences under ongoing threat, demonstrating continuous exposure, multisystem responses, moral injury, coping under strain, and professional identity strain. The study advances a conceptual model for understanding and responding to therapists under sustained threat and attests to the need for CTS-informed supervision, organizational support, and profession-specific assessment tools.

## 1 Theoretical overview and literature review

### 1.1 Continuous traumatic stress: definition and conceptual development

Continuous Traumatic Stress (CTS) describes living under an ongoing, real or perceived threat, where the source of danger is not a single traumatic event that has ended but a persistent, unresolved, and often unpredictable reality (1, 2). Unlike classical trauma models focusing on a discrete past event, CTS concerns a threat that continues to shape daily life, sense of security, and coping capability (3). Thus, CTS serves as a conceptual framework for living *under* ongoing threat, rather than responding to an event that has concluded.

CTS has emerged from the theoretical need to understand the effects of war, terrorism, political violence, and daily threat exposure, in which reality generates sustained vigilance, anxiety, and uncertainty (1, 2). In Israeli literature, CTS has been presented as a multidimensional phenomenon affecting personal, social, and communal domains (4). Recent studies have expanded the definition to ongoing domestic violence, where women experience continuous threat even after separation (5).

### 1.2 CTS vs. PTSD/CPTSD

The primary distinction between CTS and Post-Traumatic Stress Disorder (PTSD) lies in the temporal structure of the traumatic experience. PTSD is anchored in a discrete past event, whereas CTS describes an ongoing threat characterized by persistent anticipation of danger, sustained hypervigilance, and absence of closure (3, 6). A recent cross-sectional study of Palestinian undergraduates during the Gaza war found high rates of PTSD, complex PTSD, and general psychological distress, and checkpoint exposure was associated with substantially higher odds of both (7).

CTS has been theorized to extend beyond impairment in self-regulation, identity, and relationships to include the continuous presence of an external threat that continues to shape individuals’ lives (3, 8). This formulation describes experiences such as “no sense of afterward,” a “waiting room state,” and living with sequences of alerts, sirens, and therapy under persistent threat. On this account, CTS constitutes a distinct experiential axis from PTSD despite partial symptomatic overlap, a proposition the present study explores rather than tests directly.

### 1.3 Models and assessment measures of CTS

The literature characterizes CTS as a dynamic, multidimensional model. Eagle and Kaminer (1) described CTS as emerging from dangerous, unstable realities where continuous threat exposure prevents full adaptation. Nuttman-Shwartz and Shoval-Zuckerman (3) proposed a cyclical model of exposure-response-anticipation of danger, where individuals operate in a sustained loop of hypervigilance.

The CTSR scale, developed by Goral et al. (8), captures three unique factors: exhaustion/detachment, anger/betrayal, and fear/helplessness. Recent studies have validated CTSR with Lithuanian adolescents exposed to ongoing violence (9) and residing in conflict zones. Nevertheless, the instrument may not fully capture CTS complexity in diverse cultures, requiring further theoretical, cultural, and psychometric refinement.

### 1.4 Ongoing exposure of populations to threat

CTS has been widely documented in circumstances of war, terrorism, forced displacement, political violence, and social instability (4, 10–14). Recent studies have expanded the concept to include prolonged domestic violence. Hulley et al. (5) examined ongoing exposure to intimate partner violence of women following separation and found that CTS was characterized by a persistent sense of threat, sustained hypervigilance, and disruption of daily functioning, even in the absence of a single clearly defined traumatic event. The study showed that cumulative exposure to “current threat” generated distinct psychophysiological responses, calling for long-term intervention.

Similarly, Leshem et al. (14) examined continuous exposure to terrorism in Israel during the COVID-19 pandemic and found that COVID-19-related concerns mediated the association between continuous traumatic stress and depression, with perceived social support moderating this pathway. The study demonstrated the need for multidimensional assessment tools capable of capturing the cumulative effect of CTS on emotional regulation and social functioning.

Studies conducted in Israel further show that these effects extend to physical, psychosocial, family, communal, spiritual, and functional domains (4, 14). In addition to being an emotional response to threat, CTS reflects an entire way of life under conditions of threat, where survival, identity, and future orientation are continually reshaped.

### 1.5 Therapists and helping professionals under dual exposure

CTS is particularly significant for helping professionals because of their dual exposure, as civilians living under threat and as professionals exposed to their clients’ trauma narratives (15, 16). This condition, termed shared traumatic reality or double exposure, blurs the boundary between personal and professional exposure, intensifying emotional and systemic overload.

Research on therapists in various countries and settings describes a similar situation where therapists and clients share the same threatening reality, with professional and civilian exposure intertwined (17–19). The result is increased risk for secondary traumatization, burnout, compassion fatigue, and impaired professional self-efficacy (20–22). Recent studies in conflict zones have confirmed the effectiveness of CTSR in measuring the dual exposure of helping professionals, especially exhaustion and helplessness (23). Therapists described sessions interrupted by sirens, sustained emotional overload, and absence of separation between the “therapist self” and the “citizen self,” where the experience is conceptualized as occupational at one level and as traumatically-ecological at another.

### 1.6 Moral injury, secondary traumatization, and burnout

Moral injury refers to the distress arising from violations of one’s core values, feelings of betrayal, guilt, or the experience of moral failure in extreme situations (24). Therapists described feelings of guilt for interrupting treatment during sirens, a sense of betraying their patients, and disappointment in systems that failed to provide adequate support (25). These accounts are consistent with emerging literature on moral trauma, moral distress, and moral injury, expanding the understanding of distress beyond purely traumatic symptomatology.

The literature describes secondary traumatization and compassion fatigue as common responses by helping professionals who are indirectly exposed to ongoing suffering (20, 26, 27). These phenomena are characterized by diminished professional self-efficacy, emotional detachment, loss of empathy, and a sense of therapeutic failure, resulting from cumulative exposure to patients’ trauma narratives. Recent studies of youth exposed to ongoing violence suggest that CTSR may be more appropriate than PTSD to describe such circumstances, particularly regarding anger and betrayal as key moral components reflecting profound violations of values and trust (9).

In a study by Leshem et al. (28) of therapists in Ukraine, practitioners reported uncontrollable crying, emotional numbing, anger, helplessness, and a sense of professional failure, alongside diminished empathy and patience toward patients. These descriptions point to an intersection of personal emotional burden and professional strain, where therapists are required to cope with their threats and the suffering of others. Thus, CTS of therapists should be conceptualized as a complex phenomenon where ongoing trauma, professional burnout, and moral injury intersect, generating a feedback loop that exacerbates distress and challenges professional boundaries.

### 1.7 Coping strategies, resilience, and systemic implications

Alongside risk and distress, the literature describes coping resources and resilience as central protective factors for populations living under conditions of CTS. Studies identify seven primary strategies: active coping, self-care, support from family and colleagues, professional supervision, therapeutic meaning-making, volunteering, and spiritual connection, all of which contribute to emotional regulation, reduced exhaustion, and sustained functioning (29).

In the study by Leshem et al. (28), work was perceived as a source of stability, peer support as a key factor in preventing isolation, maintaining routine as a means of preserving control, connection to nature as a form of sensory regulation, and volunteering as a source of meaning. Therapists described how sharing experiences created a common language and a sense of solidarity, while supervision helped restore professional boundaries.

At the system level, there is a clear need for sustained organizational support, CTS-informed supervision, and recognition that helping professionals are not immune to the effects of prolonged exposure. Resilience is conceptualized as an individual trait and an ecological outcome derived from interactions between the individual, family, community, and institutions (4, 30). Research on CTS in Israel found that community and spiritual resources can mitigate psychosocial harm and reduce feelings of abandonment and isolation. Thus, coping with CTS should be framed as a multi-level ecological process, in which systemic support complements individual efforts and enables long-term endurance under conditions of ongoing threat.

### 1.8 Knowledge gaps and the conceptual framework of the present study

Despite significant theoretical advances, ambiguity remains in the definition of CTS, with substantial overlap between CTS, PTSD, CPTSD, secondary traumatization, and moral injury (6, 8, 24). Measurement tools such as the CTSR scale, originally developed for general adult populations (8), may not fully capture the complexity of therapeutic work under conditions of dual exposure of helping professionals. The literature reveals the need for further research on therapists who live in the same threatening reality in which they provide care, and for an integrative framework that comprises exposure, response, coping, and moral injury.

The present study addressed this gap by proposing a dynamic exposure–response–coping model for therapists that conceptualizes CTS as an organized professional-ecological experience under conditions of ongoing threat. The study proposes a framework that integrates continuous threat, multi-systemic responses, moral injury, and secondary traumatization, accounting for both individual and systemic resilience resources. The literature review leads to the following research questions: How do therapists construct the experience of CTS under ongoing threat? What distinguishes the unique components of CTS from those of classical trauma? What is the theoretical and applied significance of CTS?

## 2 Materials and methods

CTS was used as an interpretive framework rather than as a directly measured diagnostic construct. Therefore, the analysis examined whether and how participants’ narratives reflected CTS-relevant processes, without claiming that CTS was psychometrically assessed or clinically diagnosed.

### 2.1 Study design

This study employed a qualitative design using semi-structured individual interviews and one focus group to explore how frontline therapists construct and interpret continuous traumatic stress under ongoing threat.

The focus group was used to enrich, contrast, and deepen the interview data by capturing shared meanings and interactional elaboration rather than as a separate quantitative strand.

The design combined individual and collective perspectives to capture the dynamic interaction between exposure, multisystem responses, and long-term coping under ongoing security threats. Data collection took place in 2025, balancing temporal proximity to events with participants’ capacity for reflection.

### 2.2 Participants

Twenty mental health professionals participated: eight psychologists, five social workers, four psychotherapists, and three educational counselors, comprising 16 women (80%) and 4 men (20%). Twelve had primary exposure as local residents in directly affected communities in southern Israel; eight had secondary exposure as nearby clinicians treating evacuated clients. Mean age was 43.8 years, and mean clinical experience was 16.1 years (**Table 1**).

**Table 1:** Demographic and professional characteristics of participants (N = 20).

ID	Age	Gender	Profession	Experience (years)	Exposure type
P1	42	F	Psychologist	15	Primary
P2	38	F	Social Worker	12	Primary
P3	50	M	Psychotherapist	22	Primary
P4	45	F	Educational Counselor	18	Primary
P5	39	F	Psychologist	14	Primary
P6	47	F	Educational Counselor	16	Primary
P7	52	M	Educational Counselor	25	Primary
P8	41	F	Psychotherapist	13	Primary
P9	44	F	Psychologist	17	Primary
P10	48	F	Psychotherapist	20	Primary
P11	36	F	Psychologist	10	Secondary
P12	43	M	Social Worker	16	Secondary
P13	40	F	Social Worker	12	Secondary
P14	46	F	Psychologist	15	Primary
P15	37	F	Psychologist	11	Secondary
P16	49	F	Social Worker	19	Secondary
P17	35	M	Psychotherapist	9	Primary
P18	51	F	Social Worker	23	Secondary
P19	42	F	Psychologist	14	Secondary
P20	44	F	Psychologist	17	Secondary

### 2.3 Recruitment and inclusion criteria

Participants were purposively recruited by snowball sampling through professional networks and institutional ethics-approved postings in southern Israel. The inclusion criteria were: (a) direct clinical work with October 7, 2023, victims or survivors; (b) personal exposure to ongoing security threats (rocket fire, alarms); (c) a minimum of two years of professional experience. The exclusion criterion was serving in an acute crisis intervention role without ongoing therapy. Thematic saturation was reached after 12 interviews and one focus group, evidenced by pattern repetition in exposure-response-coping constructs.

### 2.4 Data collection

The interviews (45–90 minutes) and the focus group (90 minutes) were conducted by the first author in the participants’ familiar workspaces. Semi-structured guides explored exposure patterns, multisystem responses, and coping trajectories. Sessions were conducted in Hebrew, audio-recorded with consent, transcribed verbatim, and anonymized using pseudonyms. Field notes documented nonverbal cues and contextual observations.

The interview guide included open-ended questions about patterns of exposure, emotional and bodily responses, professional functioning, coping strategies, and perceived changes in therapeutic presence. The focus group guide addressed shared experiences, common themes, points of agreement and disagreement, and collective meanings of working under ongoing threat. Individual interviews were conducted between 2/2025-8/2025, and the focus group took place in April, 2025. All sessions were conducted in Hebrew, audio-recorded with consent, transcribed verbatim, and checked against the recordings for accuracy. Transcripts were analyzed in the original language; quotations translated for publication were reviewed for semantic fidelity.

The rationale for combining interviews and focus group data was to capture both private accounts and socially negotiated meanings. Themes were developed inductively and iteratively through repeated reading, coding, clustering of codes into candidate themes, and refinement through comparison between participants and data sources. Saturation (information power) was assessed by tracking the repetition of core patterns and the absence of substantially new conceptual insights in the final interviews and in the focus group.

### 2.5 Data analysis

Data underwent reflexive thematic analysis, using MAXQDA, following Braun and Clarke’s (31, 32 six-phase framework: (a) familiarization with data through repeated reading; (b) inductive semantic coding to generate initial codes; (c) deductive grouping aligned with CTS constructs, exposure-response-coping (8); (d) theme development through pattern clustering and collating; (e) theme review and refinement by peer debriefing; (f) final synthesis and write-up with illustrative quotes.

The data were analyzed using reflexive thematic analysis following Braun and Clarke. The first author read the transcripts repeatedly, conducted initial coding, and iteratively developed codes and themes. A research assistant contributed to the thematic stage through joint review of selected transcripts, reflexive discussion of codes and themes, and refinement of boundaries between closely related codes. This collaborative process was intended to deepen interpretive insight, strengthen reflexivity, and enhance analytic clarity, rather than to establish inter-rater reliability. All stages of the analysis were systematically documented to ensure transparency and traceability of analytic decisions.

### 2.6 Ethical considerations

The study received institutional review board approval from Achva Academic College, Protocol No. 121. Participants provided written informed consent after receiving guarantees of voluntary participation, confidentiality (pseudonyms used in findings, encrypted storage of data), the right to withdraw from the study at any point, and that data would be used exclusively for research. No incentives were offered. Emotional distress protocols were established, with referrals provided as needed.

### 2.7 Trustworthiness and rigor

Trustworthiness followed Lincoln and Guba’s criteria (33, 34): credibility (prolonged engagement, member checking of themes, thick descriptive quotes); transferability (purposeful diverse sampling, thick contextual description); dependability (detailed audit trail of analytic decisions); confirmability (reflexivity maintained through a positionality journal and continuous documentation of interpretive decisions).

## 3 Results

### 3.1 Overview of themes

The analysis yielded five interrelated themes that describe how frontline therapists experienced continuous traumatic stress under ongoing threat. Participants did not merely report exposure events; they described an ongoing process of anticipation, bodily vigilance, emotional depletion, professional strain, and attempts to preserve therapeutic functioning. The themes are presented below with analytic interpretation, subthemes (where relevant), and illustrative quotations that show how each theme was grounded in the data. **Table 2** summarizes the five main themes revealed by the investigation. These themes are presented as qualitatively relevant patterns rather than as prevalence estimates. .

**Table 2:** Main themes and qualitative descriptors.

Theme	Description
1. Continuous multi-channel exposure	Alarms, indirect client trauma, and work-related demands merge into a single, unbroken state of threat that collapses the boundary between home and work.
2. Polymorphic multisystem responses	Threat registers simultaneously in emotional, cognitive, somatic, and behavioral responses, with bodily anticipation of danger often preceding conscious recognition.
3. Moral injury as a unique therapist response	Interrupting care during alarms, institutional neglect, and eroding professional presence generate guilt, betrayal, and a felt violation of therapeutic values.
4. Long-term coping trajectories between burnout and renewal	Participants oscillate between depletion and partial restoration through brief rituals, professional meaning, and collegial language, without reaching stable recovery.
5. Therapist-specific secondary traumatization and professional identity strain	Repeated exposure to client narratives reshapes therapists’ worldview and erodes their sense of professional presence and identity.
Theme	Qualitative descriptor
Continuous multi-channel exposure	Ongoing overlap of home, work, and community threat.
Multisystem responses	Emotional, cognitive, somatic, and behavioral responses to continuous threat.
Moral injury	Guilt, betrayal, and disruption of professional values under ongoing threat.
Long-term coping trajectories between burnout and renewal	Oscillation between depletion, temporary restoration, and pragmatic adaptation.
Therapist-specific secondary traumatization and professional identity strain	Shifts in worldview, therapeutic presence, and professional self-understanding.

The five themes form a qualitative dynamic model in which continuous exposure is linked with multisystem responses and moral injury, alongside coping efforts and therapist-specific identity strain.

Theme 1: Continuous multi-channel exposure

This theme captures participants’ experience of living under a continuous threat environment in which alarms, indirect client trauma, media exposure, and work-related demands were no longer separate channels but part of one ongoing state of alert. Rather than describing isolated events, participants spoke about a collapsed separation between home and work, between civilian life and professional life, and between external danger and internal readiness. Two subthemes were especially visible: the collapse of temporal boundaries and the accumulation of exposure across contexts.

Participants with primary exposure, who lived in affected communities, noted the immediacy of bodily alarm and the impossibility of “switching off” even briefly. One participant said: “You finish a phone call with a client in a shelter, and then a siren is heard at home. There’s no separation in my mind, it’s the same threat from another angle.” This statement does more than describe two stressful events; it shows how the participant experienced them as one continuous threat. The phrase “no separation in my mind” reflects the central analytic point of this theme: the threat is not experienced as episodic but as constantly present and transferable between settings.

Another participant described the persistence of alertness even when there was no immediate event: “Even when it’s quiet, the sounds continue. Every distant boom, every news update returns me to emergency readiness. There’s no after phase. We’re always in a waiting room between rounds.” The excerpt illustrates the temporal logic of CTS. Quiet does not restore safety; instead, silence becomes another moment of suspended vigilance. The metaphor of a “waiting room” expresses a state of unresolved anticipation, where exposure continues psychologically even in the absence of direct danger.

A further layer of this theme was the cumulative overlap between civilian life and professional responsibility. One participant stated: “It became mainly only a professional life because twenty-four hours a day I worked and moved between the evacuation areas … and during this period my family basically managed without me.” This account shows that the boundary collapse was not only emotional but also ecological and role-based. Work, family, and civic crisis were fused into one overextended mode of functioning. For some participants, especially those with secondary exposure, the accumulation came less from direct alarms at home and more from the repeated intrusion of clients’ narratives into daily life. In both cases, however, the data indicate that exposure was experienced as layered and continuous rather than discrete.

Additional voices added further facets to this continuous exposure. Some described the automatic redistribution of attention under threat: “The body operates on autopilot: Where are the children, where are the clients, who needs a calming message? Even before I ask, how do I feel?” Others captured the absence of any clear end-point: “We hear horror stories from clients every day, and then return home to sirens; uninterrupted double trauma”.

Sustained exposure also altered affective thresholds. One participant described how proportionality itself shifted over time: “I’m more short-tempered, more impatient, and … also the proportions change. On one hand, I really appreciate things much more … and on the other, for something to excite me now or to be dramatic … people’s distress, like patients’ in the clinic, already need to cross some threshold for me to really feel that they’re miserable and that it’s hard.” This account points to a form of affective recalibration: beyond exhaustion, it reflects a restructuring of the emotional register through which both personal and professional experience is filtered.

Overall, this theme suggests that the participants’ distress was produced not by separate traumatic episodes but by the ongoing convergence of multiple threat channels that eroded the distinction between personal safety, professional presence, and everyday routine.

Theme 2: Polymorphic multisystem responses

Theme 2 shows how that exposure was registered across emotional, cognitive, somatic, and behavioral systems. In the participants’ accounts, continuous threat was encountered externally, but it also increasingly organized their internal responses. This theme reflects the multiplicity of responses that participants linked to continuous threat. Rather than describing a single symptom cluster, they spoke about emotional, cognitive, somatic, and behavioral shifts that seemed to reinforce one another. A key subtheme here was the body’s anticipation of danger, in which physical sensations such as chest pressure, headaches, muscle tension, and sleep disruption appeared even when no immediate external event was occurring.

One participant described how bodily alarm preceded conscious interpretation: “Suddenly in the middle of a normal day, a sharp pain in the chest, and then I realize it wasn’t a siren it’s only the thought about the next round.” This excerpt illustrates how threat was experienced first as bodily activation and only later as cognitive recognition. The body reacted as if danger was already present, suggesting that the participants’ response system had become sensitized to anticipation rather than to a single observable event.

Another participant noted the constant state of vigilance: “I fall asleep quickly from exhaustion but wake at every small noise and immediately check the phone and the kids. The body is always on guard.” The excerpt shows the persistence of hypervigilance day and night. Sleep does not restore safety; it merely interrupts exhaustion temporarily. The repeated checking behavior also indicates how emotional concern for family and professional responsibility merged into a single protective routine.

Participants also described how emotional responses extended into professional functioning: “There was enormous pressure to refresh the professional knowledge about intervention in trauma and bereavement and loss. At the same time, there was also a need to protect my personal and professional self because of the difficult communications about people who were killed and the horrors that were reported in the news.” This statement is analytically important because it links emotional overload to professional adaptation. The participant is describing distress but also the effort to maintain competence while being emotionally overwhelmed. The excerpt shows that the multisystem response included cognitive strain, emotional saturation, and role-related self-protection.

The subtheme of somatic distress was especially manifest in reports of chest pressure, headaches, and back tension. These bodily symptoms were not described as isolated medical complaints but as part of a wider pattern of embodied threat. The data suggest that the participants’ experience was not organized around one dominant symptom, but around a cascade of mutually reinforcing responses involving the body, emotion, cognition, and behavior.

Anger and irritability also spilled into personal relationships, pointing to how the emotional load of continuous threat extended beyond the professional domain: “I catch myself snapping at my partner or colleagues, as if the anger is at them, but inside it’s uncontrolled anger at a reality.”.

The somatic dimension appeared vividly in participants’ language. Several described the body as carrying the burden of vigilance independently of conscious awareness: “The body remembers before the mind does; headaches, chest pressure, back tension, without apparent cause.” This formulation captures a key feature of continuous threat: the body does not distinguish between active alarm and the intervals between threats. Somatic activation persists as a baseline state.

Overall, this theme demonstrates that continuous threat was experienced as a multisystem process, with bodily vigilance, emotional exhaustion, and behavioral protection functioning together rather than separately.

Theme 3: Moral injury as a unique therapist response

Theme 3 addressed the ethical and relational meaning that these responses acquired during therapeutic work. The findings suggest that distress was experienced as exhaustion, vigilance, as well as a challenge to professional values and obligations. The theme captures the ethical and relational distress that emerged when participants felt unable to remain fully present for clients while simultaneously protecting themselves and their families. The data suggest that moral injury was not a separate reaction from exposure, but a way in which exposure became professionally meaningful and emotionally painful. A subtheme here was the clash between professional duty and civilian survival, especially in moments when therapy had to be interrupted by alarms or safety procedures.

One participant expressed the experience of interruption as a moral breach: “I feel that I’m betraying my patients when I have to stop the treatment in the middle of a siren. How am I supposed to be fully present when I’m constantly thinking about my kids in the shelter?” This excerpt is analytically important because it links interrupted treatment to inconvenience as well as to a perceived violation of professional ethics and identity. The sense of betrayal operates on two levels: toward patients and toward the participant’s own professional self.

Another participant framed the lack of support as institutional betrayal: “The state abandoned us. No support, no supervision, just keep working.” Here, moral injury extends beyond the therapeutic dyad and becomes institutional, as the participant framed the lack of support as betrayal by the systems that were expected to protect professional workers. The excerpt shows that distress was both about what happened in sessions and about what was missing from them.

A further element of this theme was the fragmentation of professional presence: “My professionalism is damaged. I can’t listen like I used to, my attention is split.” This statement reflects more than fatigue; it suggests an erosion of therapeutic identity and confidence in one’s capacity to provide care. The participant’s concern is not simply emotional exhaustion but a felt reduction in professional effectiveness and presence.

These excerpts show that moral injury was tied to the impossibility of meeting both civilian and therapeutic obligations without loss. For participants with primary exposure, the tension often involved direct interruption by local danger, whereas for those with secondary exposure, it was often bound up with the inability to offer stable care to evacuated or distressed clients. In both cases, the experience was marked by guilt, betrayal, and a sense that core values had been compromised.

For participants who had been displaced from their communities, moral injury also carried an anticipatory dimension. In addition to past or present failures, the ethical breach was also about an uncertain return: “My concern is, after we return to the kibbutz, what the security situation will be, how it will affect me, how it will affect my children?” This account extends moral injury beyond the therapeutic relationship into the broader ecological fabric of the therapist’s life: the community, the family, and the future are implicated alongside the client.

Overall, this theme indicates that moral injury in frontline therapists emerges when ongoing threat disrupts the ethical conditions of care and forces repeated compromises between safety, presence, and professional commitment.

Theme 4: Long-term coping trajectories between burnout and renewal

Theme 4 turns to the ways participants attempted to continue functioning under conditions of threat. Coping did not appear as a full recovery but as an ongoing effort to create temporary stability, meaning, and professional continuity. This theme reflects the movement participants described between depletion and partial restoration. Rather than presenting coping as a simple opposition between resilience and burnout, the data suggest an oscillating process in which small routines, professional meaning, collegial language, and self-protective rituals sometimes interrupted emotional collapse but did not remove the ongoing strain. A key subtheme was the search for temporary boundaries, especially through brief routines that helped participants separate work from home life.

One participant described the need for a deliberate ritual after work: “Precisely because of the overload, I discovered I must build small end-of-day rituals, music in the car, a short walk, otherwise the trauma follows me home.” This excerpt shows that coping was not a spontaneous recovery process but a deliberate micro-practice aimed at restoring some separation between contexts. The participant did not claim to be healed; rather, the ritual functioned as a temporary buffer that prevented immediate spillover from work into home life.

Another participant described the value of meaning and professional return: “I think that functioning helped very much, the professional functioning, the very fact that I returned to what I know how to do. I was at work, I left the hotel and went to work. It helped me.” Here, the quotation illustrates coping as movement back into routine and competence. Work was not merely a source of strain; it provided structure, familiarity, and a sense of self-continuity. The analytic point is that professional functioning serves as a stabilizing resource, even as the surrounding conditions remain highly stressful.

Collegial language also emerged as a coping resource: “We have a shared CTS language with colleagues. When I say ‘autopilot,’ everyone understands exactly.” This statement shows that in addition to emotional support, colleagues also provided a shared interpretive framework that normalized distress and reduced isolation. The phrase “shared CTS language” indicates that coping was embedded in a collective professional culture rather than in individual resilience alone.

At the same time, the data suggest that this coping remained fragile. Participants repeatedly described feeling on “professional autopilot,” which points to functioning without full emotional recovery. Coping often meant continuing to work while depleted, not escaping distress entirely. For some participants with primary exposure, coping strategies centered on survival routines and immediate stabilization. For those with secondary exposure, coping more often involved maintaining therapeutic purpose, peer contact, and emotional boundaries. In both groups, the data show an ongoing tension between collapse and renewal rather than a stable return to baseline. The felt experience of this depletion was articulated directly by several participants: “There are days I feel on professional autopilot: I do what’s needed without asking if there’s space for me in all this.”.

The role of professional meaning in coping was marked by ambivalence. Meaning-making did not simply sustain participants; it also bound them to a position of ongoing exposure: “The meaning I derive from working with clients is both strength and risk: it gives purpose but anchors me to a place without real respite.” This paradox is analytically significant: the very source of professional identity that enables coping also forecloses withdrawal. For these therapists, meaning and burden were inseparable.

This theme suggests that coping under continuous threat is better understood as a trajectory of partial restoration, recurrent strain, and pragmatic adaptation than as a linear progression toward resilience. Participants’ accounts also suggest that prolonged exposure left traces that extended beyond immediate stress management.

Theme 5: Therapist-specific secondary traumatization and professional identity strain

Theme 5 examined the more enduring ways in which secondary exposure affected therapists’ sense of self, professional presence, and worldview. It concerns the ways in which client narratives and prolonged exposure reshaped how participants understood themselves, their work, and the world around them. Two subthemes were especially prominent: a widening perception of danger in the world and an erosion of therapeutic presence. These findings suggest that secondary traumatization was both emotional and identity-based, affecting how therapists experienced their professional role.

Two analytically distinct sub-patterns were apparent in the data: worldview alteration accumulated from client narratives (“the world is more dangerous”) and presence crisis (“I’m no longer the therapist I used to be”). Each sub-pattern had its own trajectory and is traced in the accounts below.

One participant described a shift in worldview: “Hearing daily about atrocities. It changes how I see the world, my trust in people. Not the same optimism.” This excerpt shows that repeated exposure to client narratives did not remain at the level of sympathetic listening. Instead, it altered the participant’s broader sense of social reality and trust. The analytic significance is that the trauma was absorbed into the therapist’s worldview, not merely into mood or stress level.

Another participant focused on the loss of therapeutic presence: “I can’t listen like before, my attention is split between treatment and life, I don’t have the same presence anymore.” Here, the quotation illustrates an erosion of the therapist’s ability to remain fully present with clients. This is not simply a complaint about tiredness; it indicates a change in the quality of therapeutic attention and in the self-experience of being a therapist. The loss of presence becomes part of the trauma itself.

A further account described blurred boundaries between the therapist self and the citizen self: “Professional boundaries are blurred, life and clients are mixed, there’s no healthy separation. The therapist I was disappeared.” This statement moves beyond secondary stress symptoms and into identity disruption. The participant feels burdened and describes a transformed professional self. The quotation shows that continuous exposure created a collapse of the boundary that usually helps sustain therapeutic role clarity.

Participants also linked this strain to the absence of systemic support: “The people who normally could have supported us were too busy … To whom does the therapist turn when he feels on the verge of breaking down?… Each one had to find out for himself.” This excerpt broadens the theme beyond individual coping and points to institutional unpreparedness. The lack of organized support intensified the strain and left participants to improvise under pressure, which in turn magnified the sense of isolation and professional vulnerability.

These quotations suggest that therapist-specific secondary traumatization in this study was marked by both worldview alteration and identity strain. The findings indicate that continuous exposure can affect what therapists feel, how they perceive the world, and how securely they can inhabit their professional role. For some participants, especially those with secondary exposure, the shift was more visible in the gradual weakening of presence and boundaries; for those with primary exposure, it was often compounded by direct personal danger. In both cases, the data show that the therapist’s role became a site of trauma-related change. The theme points to a distinctive form of strain in frontline therapists, in which secondary exposure, professional responsibility, and identity disruption are tightly intertwined.

Not all participants described identical levels of distress or loss of functioning. A smaller number reported periods of preserved therapeutic presence, strong collegial support, or moments of growth and meaning-making despite ongoing exposure. These divergent accounts suggest that CTS-related experiences varied in intensity and were shaped by support, role demands, and the balance between personal and professional exposure.

## 4 Discussion

**Figure 1:**
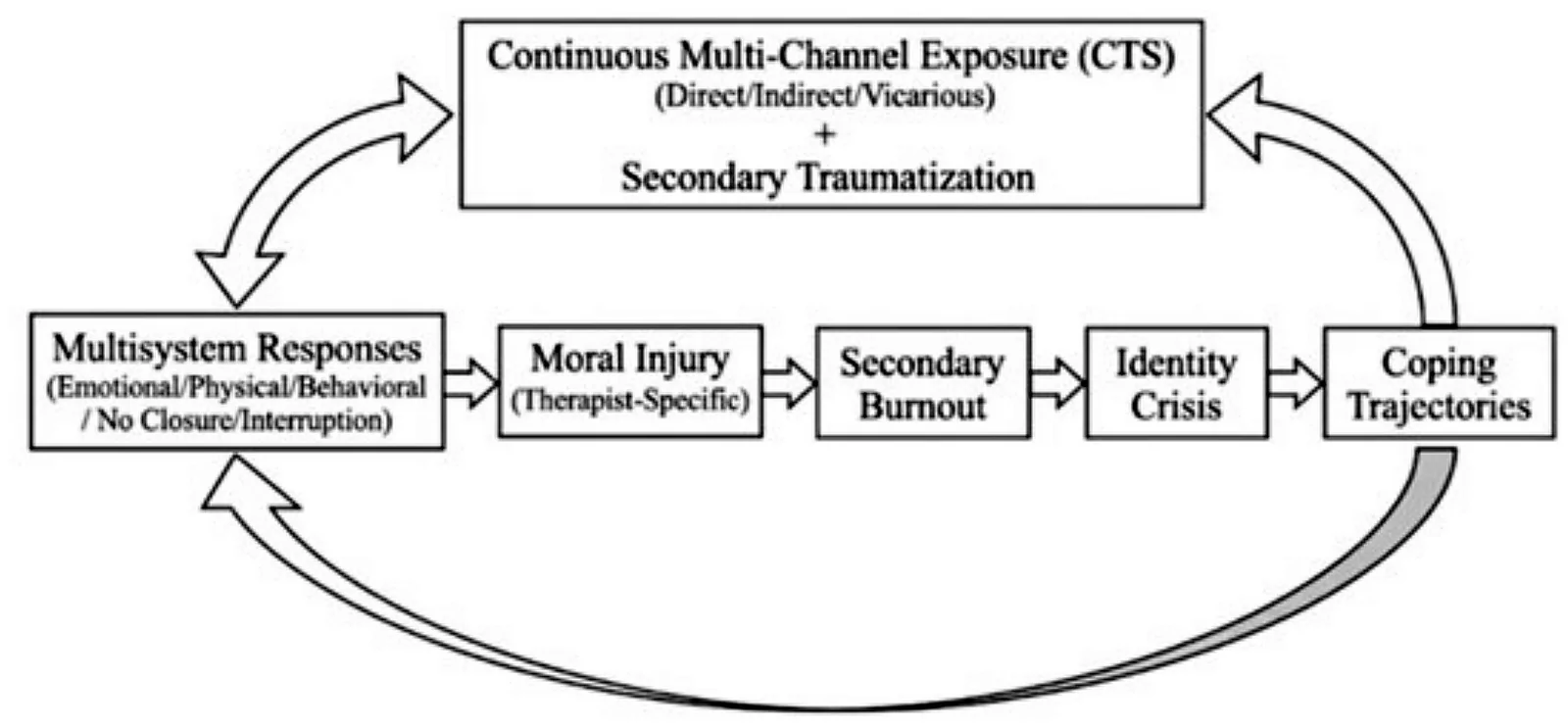
Comprehensive model of frontline therapists’ ongoing stress.

The findings are compatible with a CTS-informed interpretation of frontline therapists’ experiences under ongoing threat, but they do not establish diagnostic or phenomenological distinctiveness from PTSD or CPTSD. The study proposed a dynamic CTS model to conceptualize frontline therapists’ exposure to ongoing stress (see **Figure 1**). The model revealed five interconnected themes: continuous multi-channel exposure, multisystem responses, moral injury, long-term coping trajectories between burnout and renewal, and therapist-specific secondary traumatization and professional identity strain. Rather than indicating quantitative prevalence, these themes are presented as qualitative patterns that together capture the lived experience of ongoing threat, dual exposure, and professional strain.

These findings extend the CTSR framework of Goral et al. (8) by documenting therapist-specific manifestations during active conflict.

### 4.1 Alignment with the CTS literature

The findings align closely with core CTS constructs established in the literature: persistent threat vigilance (3), multisystem allostatic load integrating psychological, somatic, and behavioral responses (35), and the three-factor CTSR structure: exhaustion-detachment, anger-betrayal, and fear-helplessness (8). Participants’ “perpetual waiting room” metaphor precisely captures Eagle and Kaminer’s (1) seminal “no-after” state central to CTS, where danger is perceived as ongoing rather than discrete. The multi-channel exposure frequency (direct, indirect, professional) matches also Nuttman-Shwartz’s (36) dynamic exposure-response cycle model, involving the harms of cumulative threat to internal regulatory systems.

### 4.2 Relationship with PTSD and CPTSD

Rather than focusing on a discrete past event, as in PTSD (6), participants’ accounts are compatible with bidirectional symptom cycling without defined temporal boundaries, characterized by disruption between present and anticipated future threat. Moral injury elements described by participants, guilt over professional value violations, perceived betrayal of the therapeutic contract, and institutional distrust, resemble the conceptual framework of VanderWeele et al. (24), amplified here by dual exposure: both as civilians facing security threats and as therapists affected by their clients’ suffering. These accounts suggest a chronic value conflict that general-population models of moral injury may not fully capture, consistent with a need for moral processing that combines system-level acknowledgment and collegial support. These patterns are compatible with a CTS-informed account of therapists’ experience as warranting supervision and organizational support tailored to dual-role exposure, rather than as evidence that this experience is diagnostically distinct from standard trauma presentations. Some of the experiences described, like session interruptions, institutional disappointment, and split attention, may sit closer to moral distress in the Jameton (37) sense than to moral injury as defined by VanderWeele et al. (24); future quantitative research using validated measures could disentangle these constructs.

### 4.3 Contributions

Theme 5 (secondary traumatization and professional identity crisis) concerns findings that may characterize helping professionals in general, extending the findings of Dahan et al. (38) on frontline healthcare workers under rocket fire to the field of mental health. Dahan et al. (38) documented elevated CTS symptoms in medical staff facing dual exposure (direct civilian threats and occupational trauma), pointing to existential-professional aspects that are less prominent in general populations. The present study suggests possible therapist-specific manifestations such as their worldview being affected by cumulative client atrocity narratives (“the world feels more dangerous”) or erosion of therapeutic presence (“I can’t listen like before”). The ongoing exposure to threat affects therapists at the emotional as well as the professional and identity levels, impairing their ability to be therapeutically present and blurring the distinction between their identity as therapists and as citizens.

The client-mediated secondary traumatization potentially compounding direct CTS configuration suggests a feedback loop where impaired therapeutic presence may intensify moral injury (Theme 3), potentially distinguishing mental health workers from other frontline professions. Unlike acute secondary traumatic stress in disaster response (20), the present findings point to chronic identity dissolution persisting beyond the immediate crisis phases. These results suggest that dual-role traumatization (citizen and caregiver) may represent a distinctive CTS configuration warranting the development of profession-specific assessment tools and interventions targeting therapeutic self-efficacy restoration.

### 4.4 Limitations

Several limitations should be noted. The sample was small, recruited through snowball sampling, and may therefore reflect self-selection of participants who were particularly affected or especially willing to discuss distress. The study was conducted in a single country, in the course of one particular conflict, which limits transferability to other settings and periods of instability. The gender distribution was uneven, and the study did not include standardized measures of PTSD, CPTSD, or CTSR, or a comparison group. Because the design was cross-sectional and qualitative, the findings cannot support causal conclusions or diagnostic claims. Recall bias, social desirability, and the researchers’ insider positionality may also have influenced the participants’ accounts and the interpretation of data. A further limitation concerns the timing of data collection: interviews were conducted during an active conflict period (February to August 2025), and the experiences documented may reflect acute-stress or wartime-specific responses rather than CTS as a stable construct distinct from PTSD or CPTSD. Longitudinal research combining CTSR measurement (8) with a non-conflict comparison group is needed to establish the temporal stability of the findings.

### 4.5 Future directions

Future research should validate this model through longitudinal tracking, combining CTSR measures (8) with physiological biomarkers (cortisol, sleep variables). Randomized controlled trials comparing personal ritual protocols and collegial supervision with standard care could establish evidence-based interventions. Cross-cultural validation in other conflict zones (Gaza, Ukraine) can be used to assess the generalizability of the dual-role CTS. Finally, comparative studies between CTS, PTSD, and CPTSD with therapists could make diagnostic distinctions and inform the development of profession-specific clinical assessment tools.

## Data Availability

The raw data supporting the conclusions of this article will be made available by the authors, without undue reservation.
